# Current status and future prospects of particle therapy (proton beam and heavy ion therapy) in Japan—in the special issue of the *Journal of Radiation Research*

**DOI:** 10.1093/jrr/rrad034

**Published:** 2023-05-20

**Authors:** Hideyuki Sakurai, Tatsuya Ohno, Kazuhiko Ogawa

**Affiliations:** Department of Radiation Oncology, University of Tsukuba, Tsukuba, Japan; Department of Radiation Oncology, Gunma University, Maebashi, Japan; Department of Radiation Oncology, Osaka University, Osaka, Japan

From the late 1980s to 2000, the maximum annual number of cases of particle therapy in Japan was about 200, most of which formed part of clinical trials. The subsequent implementation of particle therapy for advanced medical care, approved by the Japanese government and paid for by the patient, has resulted in its increased use. Results of clinical studies on particle therapy, including prospective studies, have been published and presented. However, although the number of facilities in which this technique is performed has been increasing, few multicenter collaborative studies have been conducted.

In 2014, the Japanese Society for Radiation Oncology (JASTRO) formed a particle therapy committee to accumulate information on particle therapy in Japan towards ensuring quality at each facility. They created a treatment policy and established a system that allowed all facilities to provide the same standard of treatment. Therefore, all cases of particle therapy administered as advanced medical care in Japan have been prospectively registered and made available for analysis since 2016. In addition, to improve the level of treatment among facilities, the society conducts on-site surveys of particle therapy facilities and publicly releases the results. This has enabled detection of deviations from the standardized treatment policy and monitoring of the implementation system of the Cancer Board.

These activities have dramatically improved particle therapy research results, and in 2016, proton beam therapy was first covered by public insurance for pediatric cancer and heavy ion therapy for bone and soft tissue tumors. Furthermore, from 2018, proton beam therapy was covered by insurance for bone and soft tissue tumors, as was particle therapy for certain head and neck cancers and prostate cancer. In addition, the 2022 revision of reimbursement added coverage for liver cancer, pancreatic cancer and recurrent colorectal cancer, and the indication of heavy ion therapy for cervical adenocarcinoma was approved.

Thus, the status of particle therapy in Japan is progressing from advanced medical care to insured medical care. In this special issue of *Journal of Radiation Research*, we publish some of the results obtained in the research conducted by JASTRO. This research is the first of its kind to be published in the international literature.

